# The myth of high‐resolution liquid phase biological electron microscopy

**DOI:** 10.1002/pro.5125

**Published:** 2024-07-22

**Authors:** Edward H. Egelman

**Affiliations:** ^1^ Department of Biochemistry and Molecular Genetics University of Virginia Charlottesville Virginia USA

**Keywords:** cryo‐EM, diffusion, radiation damage, structural biology

## Abstract

Cryo‐electron microscopy (cryo‐EM) has transformed structural biology over the past 12 years, with it now being routine rather than exceptional to reach a near‐atomic level of resolution for proteins and macromolecular complexes. Samples are immobilized by vitrification and this sample can be maintained at liquid nitrogen temperatures in the vacuum of the electron microscope with negligible sublimation. Due to the low electron doses needed to avoid radiation damage, averaging over tens of thousands to hundreds of thousands of particle images is used to achieve a high signal‐to‐noise ratio. An alternative approach has been proposed where samples are at room temperature in the liquid state, maintained in the vacuum of the electron microscope by thin film enclosures that are relatively transparent to electrons while preventing evaporation of the liquid. A paper has argued that using this liquid‐phase approach, higher resolution (3.2 Å) can be achieved than using cryo‐EM (3.4 Å) when imaging and reconstructing adeno‐associated virus particles. I show here that these assertions are untrue, and that basic principles in mathematics and physics would need to be violated to achieve the stated resolution in the liquid state. Thus, high resolution liquid phase EM of macromolecules remains science fiction.

Cryo‐EM has supplanted X‐ray crystallography as the main technique being used to determine the atomic structure of macromolecular complexes. This transformation in structural biology has been based upon a number of developments. First, it has been known for >80 years that protein structure is only maintained in a fully hydrated environment (Bernal et al., 1938; Crowfoot & Riley, 1939). It was shown that the problem of imaging hydrated biological samples in the vacuum of an electron microscope could be overcome using vitrified samples that are maintained at liquid nitrogen temperatures in the electron microscope (Dubochet, 2016). Obviously, one consequence of vitrification is that the sample is relatively (but not completely, see below) immobilized on the time scale of a typical exposure. Another important consequence of cryogenic temperatures is that radiation damage is minimized, primarily due to limiting the diffusion of radiolytic products. The key role of cryogenic temperatures in minimizing radiation damage so that high resolution structure determination by electron microscopy is even possible has been known and discussed for more than 50 years (Glaeser & Taylor, 1978). This cryoprotection has also been extensively used in X‐ray crystallography (Hope, 1988). Claims of overcoming radiation damage in ambient temperature liquid phase electron microscopy were previously addressed by Glaeser (2012).

The problem of radiation damage in cryo‐EM can be further minimized by using relatively low electron doses, with the shot noise arising from the electron counting statistics overcome by averaging (Penczek et al., 1992). One of the most dramatic breakthroughs was the introduction of direct electron detectors, replacing either film or scintillator‐based CCD detectors (Kuhlbrandt, 2014). Since the frame‐rates of these new cameras are very fast, it became practical for the beam‐induced movement of the sample to be mitigated by recording movies, with motion‐correction performed by aligning successive frames before summing them (Li et al., 2013). Better microscopes and software have also played important roles in this cryo‐EM resolution revolution (Bai et al., 2015).

With this background in mind, a striking paper was published in 2021 (Jonaid et al., 2021) arguing that a higher resolution (3.2 Å) for three‐dimensional reconstruction of adeno‐associated virus (AAV) particles could be obtained using liquid phase electron microscopy than cryo‐EM (3.4 Å). Despite the standard in the field of structural biology that all such volumes need to be deposited in the Electron Microscopy Data Bank (EMDB), there were no accession codes contained in the paper for either the liquid phase or cryo‐EM volumes shown. There was, however, an AAV volume in the EMDB (EMD‐23634) deposited by these same authors on 17 March 2021, that was claimed (without any evidence) to have a resolution of 5.0 Å. On 20 March 2024 the authors linked EMD‐23634 to the Jonaid et al. paper.

There are two main ways that one may currently assess the resolution of a cryo‐EM volume. One, that has been called the “gold standard”, involves dividing the data set of particles into two non‐overlapping half sets, and generating an independent reconstruction from each half set. Comparison of these two volumes (half‐maps) in Fourier space allows one to determine the resolution where the correlation between the two volumes ceases to be significant. The convention in the field has been that this Fourier Shell Correlation (FSC) threshold should be taken as 0.143 (Rosenthal & Henderson, 2003; Rosenthal & Rubinstein, 2015). However, it has been raised that this FSC method, particularly when symmetry is imposed, is more a measure of reproducibility rather than resolution (Subramaniam et al., 2016), and that other measures should also be used in addition to the map:map FSC where the half‐maps are being compared, such as a map:model FSC when a stereochemically‐refined atomic model has been built into the density map. Given our enormous database of protein structures, we have rather large constraints that can be placed on atomic models of proteins due to prior knowledge about protein stereochemistry. Thus, one cannot simply build a protein model by placing atoms at all places of high density in the cryo‐EM map.

The Jonaid et al. paper actually provided both the map:map FSC and the map:model FSC (called *C*_ref_), reproduced here in Figure 1a. While the standard in the EMDB since 25 February 2022 has been that the deposition of the half‐maps is required, the half‐maps were never deposited for EMD‐23634 either at the time of the original submission or when it was “updated” in March 2024 to link it to the Jonaid et al. paper. However, no one has ever seen such smooth monotonic FSC curves from real data, particularly from a very limited data set containing only 280 virions (the smaller the data set, the larger the fluctuations one is expected to see in such FSC curves). For comparison, a typical FSC curve (from an icosahedral virus reconstruction at 3.3 Å resolution) is shown in Figure 1b.

**FIGURE 1:**
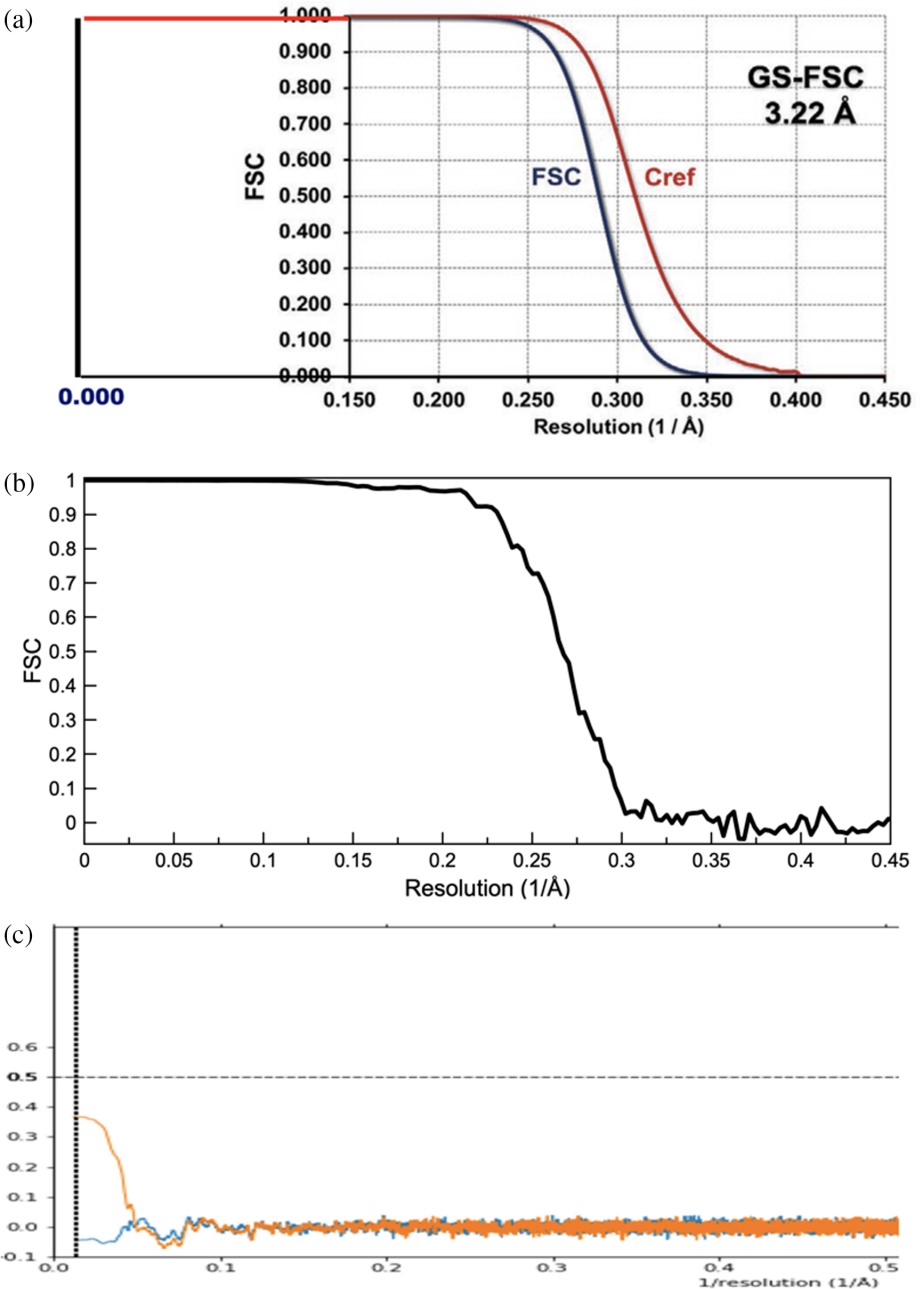
(a) The FSC/*C*_ref_ plot from Jonaid et al. (2021) begins at a spatial frequency of 0.15 (Å^−1^) rather than 0.0. I have extended the x‐scale so that it starts at 0.0, assuming that both the map:map and map:model FSC are 1.0 in this extension. The fact that the map:map FSC curve and the map:model *C*_ref_ curve started at a spatial frequency of 0.15 should have raised concerns, as these are always calculated from a spatial frequency of 0.0 out to the Nyquist frequency. The half‐maps have never been provided which does not allow the recalculation of the FSC curve shown. (b) For comparison, the map:map FSC from a typical icosahedral virus cryo‐EM structure (EMD‐42812) is shown. In contrast to the perfectly smooth FSC in (A), this plot shows the fluctuations that are always present in real FSC calculations, where the curve is never monotonic. This plot was made without any mask (excluding noise outside of the virion) and has a measured resolution of 3.4 Å. With a mask, the resolution is 3.3 Å. (c) The provided atomic model allows for a calculation of the map:model FSC (*C*_ref_) that shows no significant correlation at any spatial frequency between the map and the model, and cannot possibly be reconciled with the published curve in (A). So, one could not even say that the map has a resolution of 50 Å based upon comparison with the model. The orange curve is masked (while the blue one is not), and even then, never goes above 0.5, which is taken as the threshold for such a map:model FSC.

Simple inspection of this EMD‐23634 volume shows it to be pure noise. There are no recognizable features of protein secondary structure (such as α‐helices or β‐sheets) in this map, that should be obvious at this claimed resolution of 3.2 Å. Further, the density in the center of the volume is the same as the density where the protein capsid would be located, showing that the density does not even correspond to what would be expected for an icosahedral virion. A slice through the center of this volume is shown in Figure 2, and for comparison a similar slice through the center of a 3.4 Å resolution AAV3 cryo‐EM reconstruction (EMD‐20625) is also shown. It should be noted that EMD‐20625 is an actual virion containing a genome (Subramanian et al., 2019), and not an empty virus‐like particle (VLP). If the genuine virus reconstruction is contoured to include more density, it will show some uninterpretable central density from the icosahedral averaging of the genome, but it is clear that the peripheral capsid density will always be much stronger.

**FIGURE 2:**
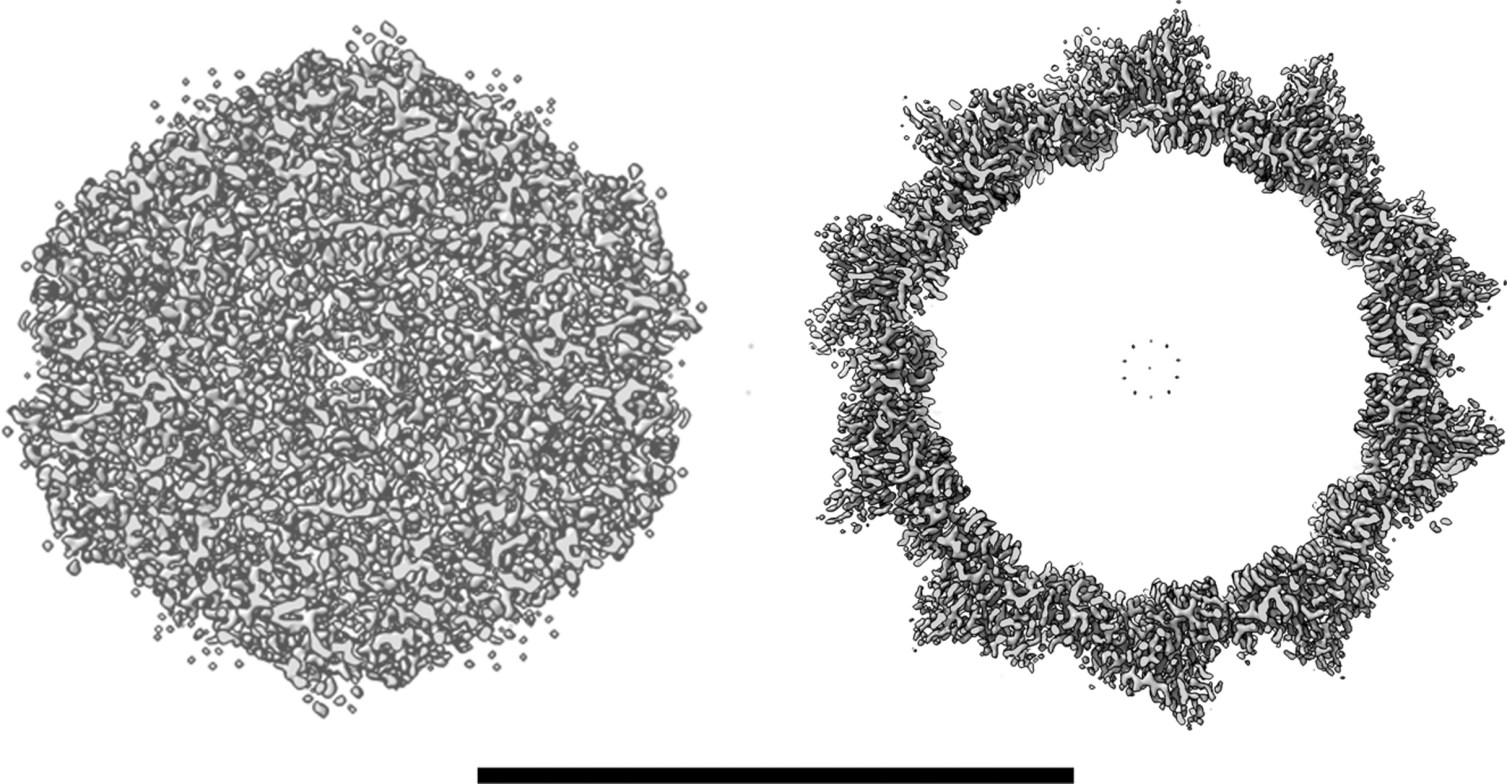
A thin central section of the AAV volume from Jonaid et al. (2021) (EMD‐23634, left) and a corresponding central section from a 3.4 Å resolution AAV3 cryo‐EM reconstruction (EMD‐20625, right). The scale bar is 250 Å.

While the half‐maps have never been provided to allow one to redo the FSC curve generated by the authors, the model used by the authors has been made available. Therefore, it is possible to redo the map:model FSC provided by the authors, the *C*_ref_ curve shown in Figure 1a. As one might expect from the visual inspection of the map, there is absolutely no correlation between the density in the map and the atomic model (Figure 1c).

Can the published results be reasonably explained? I fear not. First, let us look at what was described: 20‐s long movies of AAV particles in liquid, collected with 40 frames per second, with a total fluence of 20 electrons/Å^2^. Since the pixels were ~1.0 Å, this would mean that each frame would contain pixels each with an average exposure of 0.025 electrons. From simple Poisson statistics, and assuming no absorption of electrons (or scattering outside of the objective aperture), there would be a probability of 0.975 that a pixel would contain no electrons, and a 0.024 probability that a pixel would contain one electron. Obviously, the probabilities for containing more than one electron are infinitesimally small. For comparison, parameters from a typical cryo‐EM experiment, such as for the FSC shown in Figure 1b (Madigan et al., 2024), might involve a total fluence of ~30 e^−^/Å^2^, fractionated into 24 frames, so in each frame there would be an expectation of ~1.25 electrons/Å^2^. It was stated in Jonaid et al. that the frames were then motion‐corrected to compensate for the large translations of the virions over the course of the 20 s movies. There are two great problems with this. One is that it would be absolutely impossible to align particles between individual frames when there is a 97.5% probability that each pixel in a frame contains no signal at all. It might be imagined (although this was never stated) that individual frames were not aligned, but rather sets of frames. But if one binned together 40 frames to have an expected signal of 1.0 electron/pixel, one would be averaging the translation of a virion over 1 s of Brownian motion. It cannot be imagined that the resultant average would be blurred by less than ~3 Å.

In fact, one does not need to imagine, and can easily estimate the diffusion coefficient for AAV under the conditions used. Assuming that the temperature was 293 K, the diameter of a spherical AAV is 28 nm, and the viscosity of the buffer was 1 centipoise, one gets a diffusion coefficient (*D*) of 7.7 μm^2^/s. This estimate is quite consistent with extrapolating from experimental values measured for particles of diameters 100, 200 and 500 nm (Hedde, 2021). This diffusion coefficient would mean that over 1 s, the root mean squared displacement of an individual AAV particle would be ~6.8 μm (using msd = 6tD for three‐dimensional diffusion). If one imagines that the diffusion was constrained to two dimensions, such as from a virion being trapped in a very thin film of liquid, the root mean squared displacement over 1 s would be 5.5 μm. Further, over the time taken to collect one frame (25 ms) the rmsd of an AAV would be ~1.1 μm for three‐dimensional diffusion, and ~0.9 μm for two‐dimensional diffusion. Motion correction can compensate for the translation of particles between frames, so that the blurring due to translation is removed from the average of these frames. But motion correction cannot compensate for or eliminate the blurring due to motion occurring within the frame.

The second problem is that motion correction can be accomplished at relatively low resolution to overcome rigid‐body translations of an object (Li et al., 2013). But motion correction of frames cannot overcome rotational motions, that would be huge for small virions in solution that are not anchored to any substrate. Further, the rotational motions of a basically spherical icosahedral capsid would not even be evident until one reached a rather intermediate resolution. At best the reconstructed volume with icosahedral symmetry imposed should look like a rotationally averaged shell of density with a relatively hollow core. Since the volume does not even show such a hollow core (Figure 2), attempts to reasonably explain what was done fail.

Let us look further at the Brownian motion (Einstein, 1906) expected in liquid. The Jonaid et al. paper describes AAV particles “migrating in liquid”, “slowly moving or diffusing in liquid”, and “dynamic movements of AAV in liquid”. The authors stated: “Thin liquid layers contained static particles exhibiting less motion. Thicker layers (*t*/*λ* = 1.0) contained virus particles that migrated more readily”. It was stated that the data used in the paper for the AAV structure and movie came from thick liquid samples. But the actual images shown in the paper are completely inconsistent with the expected Brownian motion of particles in liquid. Supplementary Figure 6a in Jonaid et al. shows frames from a single movie at 5, 10 and 20 s. These same frames are also shown in Jonaid et al. Figure 4 which describes “AAV diffusion in plane” over the course of the 20 s exposure. But if one aligns the image at 20 s with the one at 5 s, it can be seen that there is no motion of individual virus particles. Rather, the entire field of fixed particles is uniformly moving over 15 s through a distance of ~210 Å. This is simply drift of the stage, and has nothing to do with diffusion in liquid. So, the statement in the paper that this movie shows “AAV in solution diffusing within 20‐s” is clearly untrue (Figure 3).

**FIGURE 3:**
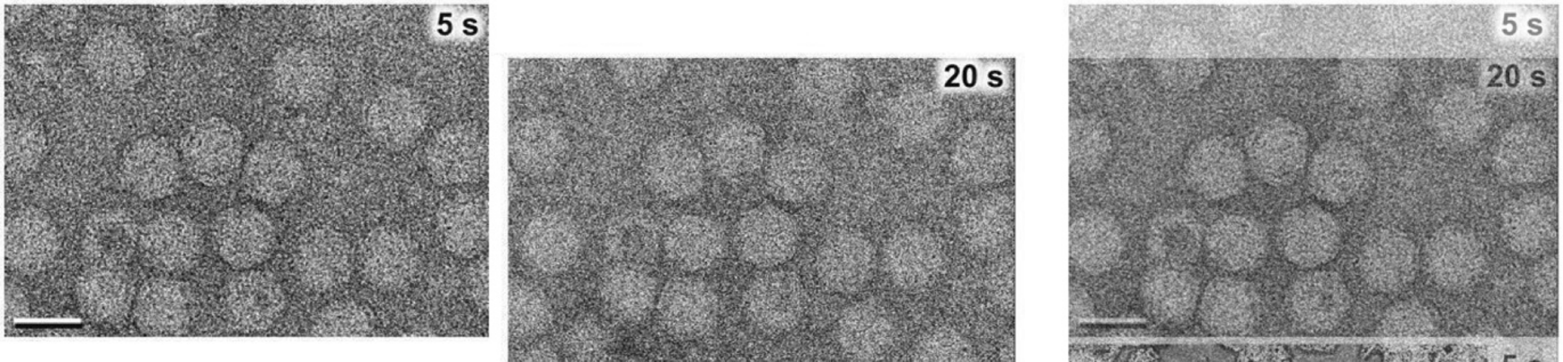
The images of a field of AAV particles at 5 and 20 s from Jonaid et al. are compared. On the left is the image at 5 s, taken as the reference. In the center, the image at 20 s has been translated by ~210 Å so as to align with the image at 5 s. On the right, the 20 s shifted image (with an opacity of 50%) has been superimposed on the 5 s image. This shows that the differences between the two images can be explained by a drift of the stage (with a rate of ~14 Å/s), and the differences between the two are completely inconsistent with any Brownian motion of the particles in liquid.

What would one expect for actual Brownian motion over 15 s? Using the estimated diffusion coefficient (*D*, above), the rms displacement of individual virions would be ~26 μm for three‐dimensional diffusion and ~21 μm for two‐dimensional diffusion after 15 s, while the frame size is only 0.4 × 0.3 μm. Thus, the vast majority of particles imaged at 5 s would no longer be in the field of view after 20 s.

The results of Jonaid et al. (2021) have been republished in a number of reviews (DiCecco et al., 2022; Jonaid et al., 2022; Kelly et al., 2022) and cited in other papers. For example, it was stated of this work (Lyu et al., 2023): “A spatial resolution comparable to cryo‐EM was achieved, as well as the tracking of dynamic conformational change of viruses, suggesting the major benefit and great potential of the liquid imaging method.” Unfortunately, the results in Jonaid et al. (2021) are unbelievable, and do not demonstrate any great potential for imaging macromolecules or macromolecular complexes by liquid phase electron microscopy. Simple considerations from physics and mathematics undercut any possibility that liquid phase electron microscopy can yield any high resolution structures of individual proteins or assemblies (Berry et al., 2023; Jonaid et al., 2021). Thus, while the prospect of using EM to observe at high resolution the dynamics of macromolecules and macromolecular assemblies in solution is tantalizing, the unfortunate reality is that everything we know about Brownian motion and radiation damage make this impossible at the present time.

## AUTHOR CONTRIBUTIONS

**Edward H. Egelman:** Conceptualization; writing – original draft; investigation.
